# Rare Adverse Events Associated with BNT162b2 mRNA Vaccine (Pfizer-BioNTech): A Review of Large-Scale, Controlled Surveillance Studies

**DOI:** 10.3390/vaccines10071067

**Published:** 2022-07-02

**Authors:** Shin-Jie Yong, Alice Halim, Michael Halim, Abbas Al Mutair, Saad Alhumaid, Jehad Al-Sihati, Hawra Albayat, Mohammed Alsaeed, Mohammed Garout, Reyouf Al Azmi, Noor Aldakheel, Abeer N. Alshukairi, Hani A. Al Ali, Adel A. Almoumen, Ali A. Rabaan

**Affiliations:** 1 Department of Biological Sciences, School of Medical and Life Sciences, Sunway University, Selangor 47500, Malaysia; 2 Shanghai Medical College, Fudan University, Shanghai 200032, China; alicehalim95@gmail.com; 3 Department of Biomedical Science, School of Science, Engineering and Environment, University of Salford, Greater Manchester, Salford M5 4WT, UK; michaelhalim1000@gmail.com; 4 Research Center, Almoosa Specialist Hospital, Al-Ahsa 36342, Saudi Arabia; abbas.almutair@almoosahospital.com.sa; 5 College of Nursing, Princess Norah Bint Abdulrahman University, Riyadh 11564, Saudi Arabia; 6 School of Nursing, Wollongong University, Wollongong 2522, Australia; 7 Nursing Department, Prince Sultan Military College of Health Sciences, Dhahran 33048, Saudi Arabia; 8 Administration of Pharmaceutical Care, Al-Ahsa Health Cluster, Ministry of Health, Al-Ahsa 31982, Saudi Arabia; saalhumaid@moh.gov.sa; 9 Gastroenterology Section, Internal Medicine Department, King Fahad Specialist Hospital, Dammam 31311, Saudi Arabia; jehadalsaihati@gmail.com; 10 Infectious Disease Department, King Saud Medical City, Riyadh 7790, Saudi Arabia; hhalbayat@gmail.com; 11 Infectious Disease Division, Department of Medicine, Prince Sultan Military Medical City, Riyadh 7790, Saudi Arabia; mohalsaeed@live.com; 12 Department of Community Medicine and Health Care for Pilgrims, Faculty of Medicine, Umm Al-Qura University, Makkah 21955, Saudi Arabia; magarout@uqu.edu.sa; 13 Infection Prevention and Control, Eastern Health Cluster, Dammam 32253, Saudi Arabia; reyouf.azmi@kfsh.med.sa; 14 Microbiology Laboratory, Dammam Regional Laboratory and Blood Bank, Dammam 31411, Saudi Arabia; noaldakheel@moh.gov.sa; 15 Department of Medicine, King Faisal Specialist Hospital and Research Center, Jeddah 21499, Saudi Arabia; abeer.alshukairi@gmail.com; 16 College of Medicine, Alfaisal University, Riyadh 11533, Saudi Arabia; 17 Pediatrics Department, Maternity & Children Hospital, Dammam 32253, Saudi Arabia; halali@moh.gov.sa (H.A.A.A.); adelalmomen@yahoo.com (A.A.A.); 18 Molecular Diagnostic Laboratory, Johns Hopkins Aramco Healthcare, Dhahran 31311, Saudi Arabia; 19 Department of Public Health and Nutrition, The University of Haripur, Haripur 22610, Pakistan

**Keywords:** adverse event, BNT162b2, COVID-19, mRNA vaccine, real-world surveillance, SARS-CoV-2, vaccine safety

## Abstract

Given the increasing anti-vaccine movements erroneously touting vaccine danger, this review has investigated the rare adverse events potentially associated with BNT162b2 (Pfizer-BioNTech), an mRNA vaccine against the severe acute respiratory distress syndrome coronavirus 2 (SARS-CoV-2). Only real-world surveillance studies with at least 0.1 million BNT162b2-vaccinated participants and one unvaccinated control group were selected for review. A total of 21 studies examining the potential association of BNT162b2 with cardiovascular, herpetic, thrombotic or thrombocytopenic, neurological, mortality, and other miscellaneous rare adverse events were described in this review. Only myocarditis is consistently associated with BNT162b2. An unclear direction of association was seen with stroke (hemorrhagic and ischemic), herpes zoster, and paresthesia from BNT162b2, which may require more studies to resolve. Fortunately, most surveillance studies detected no increased risks of the remaining rare adverse events reviewed herein, further reassuring the safety of BNT162b2. In conclusion, this review has concisely summarized the current rare adverse events related and unrelated to BNT162b2, arguably for the first time in sufficient depth, to better communicate vaccine safety to the public.

## 1. Introduction

The World Health Organization (WHO) has named vaccine hesitancy as one of the top 10 threats to global health [1]. This fact is not surprising given the progressive rise in anti-vaccine movements worldwide, especially during the coronavirus disease 2019 (COVID-19) pandemic, erroneously claiming that vaccines are unsafe, thus decreasing vaccine uptake in some countries [2,3]. COVID-19 is caused by severe acute respiratory syndrome coronavirus 2 (SARS-CoV-2), a highly infectious respiratory virus [4]. Therefore, understanding and communicating vaccine safety are necessary tasks for the benefit of global and public health [5,6].

The gold-standard method of determining the safety and efficacy of a vaccine or drug in humans is through randomized controlled trials (RCTs). RCTs randomize participants into the experimental or control groups, which account for the countless variables that could differ between individuals. Randomization, thus, ensures that the results seen are strictly due to the experimental intervention, allowing the establishment of cause-and-effect [7,8]. Owing to RCTs, several vaccines (e.g., Pfizer-BioNTech BNT162b2, Moderna mRNA-1273, Johnson & Johnson Ad26.COV2.S, AstraZeneca-Oxford ChAdOx1n, and Novavax NVX-CoV2373) have been confirmed as safe and effective at preventing symptomatic and severe COVID-19 [9,10,11,12,13]. Although these RCTs were underpowered to detect differences in COVID-19 mortality rate, other observational studies have consistently and strongly indicated that COVID-19 vaccines do prevent fatal COVID-19 [14,15,16].

However, outside of the RCT in the general population, the generalizability of RCT results is debatable [8]. RCTs have strict participant inclusion and exclusion criteria to ensure that results are more readily replicable. For example, the BNT162b2 RCT included ≥1 6-year-olds who were healthy or had stable medical conditions and excluded immunocompromised individuals [9]. The ChAdOx1n RCT only included healthy adults aged 18–55 years [12]. Participants in the Ad26.COV2.S RCT were also mostly healthy 18–59-year-old adults with no medical conditions associated with increased risk of severe COVID-19 [10]. Furthermore, these COVID-19 vaccine RCTs have about 15,000–30,000 participants, which is not huge enough to detect very rare events. For instance, detecting an adverse event with the incidence rate of 1 in 10,000, 2 in 10,000, and 3 in 10,000 with *p* < 0.05 require at least 30,000, 48,000, and 65,000 participants, respectively [17].

Hence, post-RCT (also known as phase IV or post-marketing) surveillance studies on COVID-19 vaccines are necessary to monitor vaccine safety and effectiveness in the real world, beyond the controlled settings in RCTs [17,18]. As covering both vaccine safety and effectiveness would be too extensive, this review paper will focus on vaccine safety, particularly the Pfizer-BioNTech BNT162b2 mRNA vaccine (also known as Comirnaty), the first COVID-19 vaccine that the Food and Drug Administration (FDA) approved [19].

## 2. Methods

Searching PubMed for the keywords, “(nation* OR surveillance OR cohort OR case series) AND (safe* OR risk OR adverse event) AND (BNT162b2 or Comirnaty),” returned 664 papers as of 14 May 2022, of which 21 are reviewed herein after applying the eligibility criteria of: (i) a sample size of least 0.1 million participants vaccinated with BNT162b2; (ii) the involvement of unvaccinated control group, either self-control or cohort control; and (iii) the investigation of at least one adverse event that is not a common vaccine side effect like fever, chills, muscle and joint pain, fatigue, headache, or injection site pain [9,20,21].

Anaphylaxis was not examined in this review because it is a known rare adverse event associated with vaccines in general, rather than BNT162b2 specifically [22,23]. Studies using background incidence rates as a control or reference group were excluded due to the high risks of potential pandemic-related confounders. Studies examining specific subgroups of populations such as immunocompromised or cancer patients, pregnant women, nursing home residents, or patients with specific diseases (e.g., diabetes, cystic fibrosis, or kidney failure) were also excluded due to limited generalizability to the general population. Characteristics of the surveillance studies are summarized in Table 1, with their risk details described in the subsequent sections.

## 3. Results

### 3.1. Cardiovascular Adverse Events

Among the studies that investigated potential BNT162b2 vaccination-related cardiovascular events (Table 2), only myocarditis (or myopericarditis) is consistently associated with BNT162b2 in six out of six studies from the USA, UK, Israel, Denmark, Finland, Norway, and Sweden [25,28,31,33,37,39]. Based on these studies, the general risk of myocarditis within 30 days of receiving a dose of BNT162b2 ranges from a 2–4-fold increase compared to the unvaccinated group, with an excess of 0.3–3 cases per 0.1 million people. Such risk is mainly reported in younger males after the second dose, that is, a 3–9-fold increase, with an excess of up to 14 cases per 0.1 million people.

Other cardiovascular events have also been occasionally associated with BNT162b2. For example, Husby et al. [28] found a 49% and 59% lower risk of cardiac arrest or death among BNT162b2 and mRNA-1273 recipients, respectively, compared to the unvaccinated group in Denmark. Lai et al. [34] have also reported a 42% diminished risk of overall cardiovascular diseases (i.e., heart failure, microangiopathy, stress cardiomyopathy, arrhythmia, coronary artery disease, or carditis) in BNT162b2-vaccinated than unvaccinated individuals in Hong Kong. Likewise, Whiteley et al. [44] found a 10% and 23% decreased risk of ischemic and hemorrhagic stroke, respectively, in BNT162b2 recipients less than 70 years old compared to unvaccinated individuals in the UK. Such a cardioprotective association was also present in the ≥70 year-old subgroup, but their relative risk overlapped substantially with that of the negative control (i.e., lower limb fracture), indicating that certain confounders were at play [44].

However, certain studies showed the reverse pattern. Patone et al. [38] and Hippisley-Cox et al. [27] found that BNT162b2 recipients had a 24% (60 excess cases per 10 million people) and 6% (240 excess cases per 10 million people) elevated risk of hemorrhagic and ischemic stroke, respectively, compared to the unvaccinated in UK Patone et al. [38] further noted that BNT162b2-associated increased risk of hemorrhagic stroke was only significant in females and not males for unclear reasons. Curiously, the pro-hemorrhagic stroke association of BNT162b2 was rendered non-significant when Patone et al. [38] limited the analyses to Scotland residents only; however, the fewer number of hemorrhagic stroke cases in Scotland might have limited the statistical power to detect very rare events. Other studies from Scotland [41], France [30], Israel [25], and the USA [33] also found no significant associations between BNT162b2 and hemorrhagic or ischemic stroke, myocardial infarction, or arrhythmia (Table 2). Ergo, the inconsistent association between BNT162b2 and cardiovascular diseases except myocarditis warrants further research to clarify.

More crucially, COVID-19 (even when mild) or SARS-CoV-2 infection has been strongly associated with increased risks of long-term cardiovascular diseases (e.g., stroke, dysrhythmias, ischemic heart diseases, and heart failure) at up to one-year follow-up in several large cohort studies [45,46,47,48,49], as reviewed in Yong and Liu [50] as the medical or clinical sequelae subtype of the post-COVID-19 syndrome. Overall cardiovascular risks are, therefore, much higher from COVID-19 than BNT162b2.

### 3.2. Herpetic Adverse Events

Owing to the uptick in herpes zoster cases in national surveillance systems, Wan et al. [43] performed a population-based surveillance study in Hong Kong and discovered over a 5-fold increased risk (7 excess cases per million doses) of herpes zoster within 28 and 14 days of the first and second BNT162b2 dose, respectively, compared to unvaccinated controls. Patients with post-BNT162b2 herpes zoster had a mean hospitalization period of 3.8 days, of which 7.4% had recurrent hospitalization due to herpes zoster [43]. In Israel, Barda et al. [25] also noted a 43% heightened risk of herpes zoster among BNT162b2 recipients compared to non-recipients. However, two other surveillance studies from Israel [37] and the USA [26] found no significant association between BNT162b2 and herpes zoster. Hence, the link between BNT12b2 and herpes zoster remains ambiguous (Table 3). For herpes simplex virus infection, only Barda et al. [25] have examined it, finding no significant association with BNT162b2.

Before the COVID-19 pandemic, case reports of herpes zoster occurring after vaccinations (e.g., influenza and hepatitis) have been published, although no convincing link was established [51,52]. Herpes zoster is a cutaneous infection from varicella-zoster virus (VZV) reactivation, which typically manifests during episodes of immunosuppression. The transient downregulation in VZV-specific T-cell immunity to compensate for SARS-CoV-2-specific T-cell responses during vaccination or natural infection may, thus, allow the opportunistic VZV to reactivate [53,54]. While herpes zoster is usually mild and acute, it further increases the risks of chronic neuralgia and stroke [55,56]. Therefore, further research into the inconclusive association between BNT162b2 and herpes zoster is highly encouraged.

### 3.3. Thrombotic or Thrombocytopenic Adverse Events

It was only in March 2021 that vaccine-induced thrombotic thrombocytopenia (VITT) was first coined and acknowledged as a rare, potentially fatal complication of ChAdOx1 (AstraZeneca-Oxford, England, U.K.) and Ad26.COV2.S (Johnson & Johnson, New Jersey, U.S.) adenoviral DNA vaccines against COVID-19. VITT pathophysiology is suspected to stem from anti-platelet factor 4 antibodies, probably generated in response to adenoviral antigens present in the vaccine, which consequently trigger signaling cascades culminating in massive platelet activation [57,58].

As the BNT162b2 mRNA vaccine uses lipid nanoparticles as carrier vehicles instead of an adenoviral vector, it is not known to be associated with VITT. Multiple surveillance studies from Israel [25], Scotland [36,41], France [30], Denmark [29], Hong Kong [34], the UK [24,27,32,38], and the USA [33] noted no significant association between BNT162b2 and VITT-related adverse events, such as cerebral venous sinus thrombosis (CVST), splanchnic vein thrombosis, deep vein thrombosis (DVT), pulmonary embolism, venous and arterial thromboembolism, immune thrombocytopenia, disseminated intravascular coagulation, and subarachnoid hemorrhage (Table 4).

Intriguingly, BNT162b2 has been occasionally associated with anti-thrombotic effects. In the UK, Whiteley et al. [44] found about 18–50% reduced risks of DVT, pulmonary embolism, arterial events, and mesenteric thrombosis in BNT162b2-vaccinated than unvaccinated individuals. Barda et al. [25] from Israel calculated 52% (2.9 fewer cases per 100,000 people) and 54% (2.2 fewer cases per 100,000 people) diminished risk of intracranial hemorrhage and thrombosis events (arterial or venous), respectively, in BNT162b2 recipients compared to unvaccinated individuals. Barda et al. [25] suspected that the anti-thrombotic association of BNT162b2 might be an indirect effect of the vaccine protecting against undiagnosed SARS-CoV-2 infection in the study. After all, SARS-CoV-2 is known to exhibit pro-thrombotic effects as part of the COVID-19 pathophysiology [59,60]. Alternatively, unmeasured confounding may be involved, as Whiteley et al. [44] have suggested based on their findings of lower risk of negative control (i.e., lower limb fracture; supposedly unrelated to the vaccine) in BNT162b2 recipients.

### 3.4. Neurological Adverse Events

Neurological diseases are diverse, many of which have been studied for their potential association with BNT162b2. As neurovascular or cerebrovascular diseases (e.g., stroke and CVST) were covered in the previous sections, they will not be discussed herein. For Bell’s palsy, multiple surveillance studies from Israel [25,40], Hong Kong [34,42], USA [33], UK [35,38], and Spain [35] have noted no significant association with BNT162b2. Some of these studies further found no significant association between BNT162b2 and other neurological adverse events, namely paresthesia, seizure, vertigo, myelitis, encephalitis, encephalomyelitis, meningitis, myasthenic disorder, and Guillain-Barré syndrome [25,33,34,38] (Table 5). 

Thus far, the only study that reported BNT162b2 to be associated with non-cerebrovascular neurological disease is Shasha et al. [40] from Israel, where BNT162b2-vaccinated individuals had a 21% (39.5 excess cases per 10,000 person-year) increased risk of numbness or tingling sensation, otherwise known as paresthesia. Oddly, however, another research from Israel found no association between BNT162b2 and paresthesia [25]. Differences in diagnostic or ascertainment tools, sample sizes, or statistical methods may explain such discrepancies. Shasha et al. [40] et al. further speculated that such symptoms of numbness and tingling might be due to anxiety-related factors or possibly other confounders. Therefore, surveillance studies examining the relationship between BNT162b2 and paresthesia are still lacking.

### 3.5. Mortality Adverse Events

Mortality is another reliable indicator of vaccine safety since it captures nearly all aspects of fatal adverse events regardless of disease types. One study from Denmark found no significant differences in death rates between BNT162b2-vaccinated and unvaccinated individuals [29]. However, another Danish study reported a 49% decreased risk of cardiac arrest or death in BNT162b2 recipients compared to non-recipients [28]. As both Danish studies used the same database, differences in endpoint (all-cause vs. cardiac mortality) or study design (self-controlled vs. matched control) may explain their discrepant findings. Hviid et al. [29] further speculated that the infrequent administration of COVID-19 vaccines to terminally ill patients might contribute to the lower mortality rate in BNT162b2 recipients in their study.

One surveillance study from the UK calculated 76% and 81% reduced risks of death among BNT162b2 vaccinees of <70 and ≥70 years old, respectively, compared to their respective unvaccinated controls [44]. Furthermore, a study from the USA found about a 60% decreased risk of non-COVID-19 mortality among BNT162b2-vaccinated than unvaccinated individuals, but the authors speculate that the healthy vaccinee effect may be involved [61] (Table 6). A healthy vaccine effect is a form of bias where healthy individuals are more likely to get vaccinated than unhealthy or frail individuals with pre-existing comorbidities [62]. Indeed, it has been critiqued that mortality is a non-specific endpoint that may exaggerate vaccine benefits in observational studies [63]. Nevertheless, the work of Xu et al. [61] suggests an intriguing possibility: BNT162b2 may not just prevent fatal COVID-19 but other fatal diseases as well, perhaps through a yet-to-be-discovered mechanism.

### 3.6. Other Miscellaneous Adverse Events

Other miscellaneous adverse events have also been studied for their potential association with BNT162b2 (Table 7). For example, Barda et al. [25] from Israel found that BNT162b2 was associated with elevated risks of appendicitis (1.4-times) and lymphadenopathy (2.4-times), but lowered risks of anemia (1.21-times) and acute kidney injury (1.56-times). In contrast, Klein et al. [33] from the USA noted no association between BNT162b2 and appendicitis. Moreover, Lai et al. [34] from Hong Kong showed that BNT162b2 recipients had 1.42-times reduced risks of hepato-renal (acute kidney injury, acute liver injury, and pancreatitis) and respiratory (acute respiratory distress syndrome) diseases. Similar to Xu et al. [61], Lai et al. [34] also suspected that the healthy vaccine effect may have confounded their vaccine protective findings.

Other adverse events, that is, neutropenia, lymphopenia, thyroiditis, type 1 diabetes, arthritis, Kawasaki disease, erythema multiforme, chilblain, uveitis, rhabdomyolysis, syncope, and narcolepsy were not associated with BNT162b2, at least not in the surveillance studies identified in this review [25,33,34] (Table 7).

## 4. Discussion

**Figure 1:**
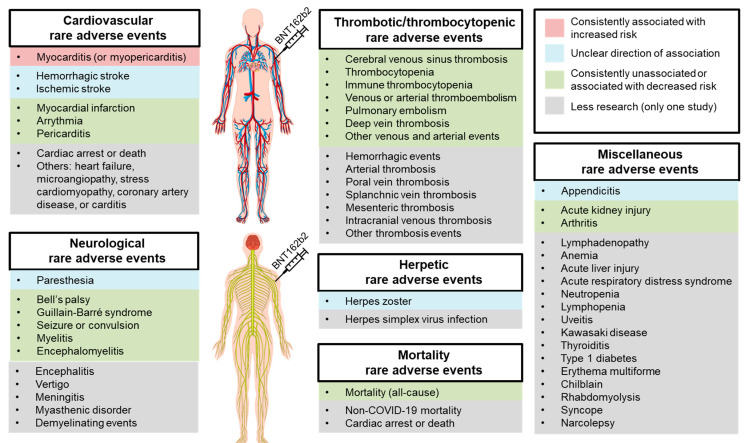
Rare adverse events potentially associated with the BNT162b2 mRNA vaccine (Pfizer-BioNTech). Notes: Red box denotes consistent association with increased risk; blue box denotes unclear direction of association (mixed signals); green box denotes no association or consistent association with decreased risk; grey box denotes less research (only one study). (The human anatomy figures were licensed from Freepik.com (accessed on 18 May 2022)).

Based on the large-scale, controlled surveillance studies reviewed herein, the only rare adverse event strongly and consistently (in six out of six studies) associated with BNT162b2 is myocarditis (or myopericarditis) at 2–4-fold increased risk compared to no vaccine (Table 2; Figure 1). Despite that, myocarditis risk from SARS-CoV-2 infection or COVID-19 is much greater, assuming equal exposure for both groups. For example, Barda et al. [25] and Patone et al. [39] calculated an 18.3-fold and 9.8-fold heightened risk of myocarditis, respectively, in SARS-CoV-2-infected than uninfected individuals. Such respective risks were only 3.2-fold and 3.4-fold when comparing BNT161b2 to no vaccine [25,39].

Post-BNT162b2 myocarditis was primarily reported in young males [25,31,33,37]. Although the precise reason remains unclear, researchers suspect that the pro-inflammatory effects of testosterone might predispose young males to post-mRNA vaccine myocarditis; in contrast, estrogen is known to be anti-inflammatory [64]. However, two (out of six) studies found a contrasting pattern, where increased myocarditis risk from BNT162b2 was limited to females only for uncertain reasons; but myocarditis risk following mRNA-1273 (Moderna) was still higher in males than females [28,39].

Indeed, myocarditis risk is typically higher following mRNA-1273 than BNT162b2 vaccination. Husby et al. [28] showed that myocarditis risk is 3–4-fold higher in recipients of mRNA-1273 than BNT162b2. Similarly, Patone et al. [39] calculated a 21-fold increased risk of myocarditis (15 excess cases per million people) in mRNA-1273 recipients compared to unvaccinated individuals; this number was only 3-fold (3 excess cases per million people) for BNT162b2 recipients. Furthermore, Karlstad et al. [31] reported an 8.6-fold elevated risk of myocarditis (5 excess cases per 0.1 million people) in mRNA-1273 recipients versus only 2-fold in BNT162b2 recipients when compared to their respective unvaccinated control group. Such differences could be due to the higher vaccine dosage used in mRNA-1273 than BNT162b2 (100 vs. 30 µg), making mRNA-1273 more durable against COVID-19 yet more immunogenic [65,66].

Fortunately, post-mRNA vaccine myocarditis is typically acute and mild. In the study of Patone et al. [39], the median hospitalization period for post-mRNA vaccine myocarditis was 3–4 days. In another surveillance study, only 4.5% and 2% of post-mRNA vaccine myocarditis cases developed heart failure and died, respectively, at 28-day follow-up [28]. Similarly, one uncontrolled cohort study detected 54 myocarditis cases among 2.5 million BNT162b2 recipients, of which 76% cases were mild, 22% were moderate, and 2% were severe [67]. In a meta-analysis of 39 cohort or observational studies comprising 129 cases of post-mRNA vaccine myocarditis, 81% of cases fully recovered within the first week, 7% required intensive care unit admission, and 1.6% died [68].

While the precise mechanism driving post-mRNA vaccine myocarditis remains obscure, researchers suspect that cardiomyocyte inflammation and cytotoxicity may result from (i) mRNA immune reactivity, (ii) molecular mimicry between spike protein and cardiac self-antigens, or (iii) mRNA vaccine-induced spike protein expression [64,69]. Interestingly, one mouse study showed that intravenous (but not intramuscular) injection of mRNA vaccine resulted in (i) spike protein expression on cardiomyocytes, (ii) increased serum levels of troponin and pro-inflammatory mediators, and (iii) histopathological changes indicative of myocarditis [70]. Therefore, researchers suspect accidental leakage of traces of mRNA vaccine into the veins during intramuscular injection might induce myocarditis. As follows, aspiration, the act of withdrawing the syringe plunger before injection to avoid vessel puncture, may reduce the risk of myocarditis in mRNA vaccine recipients [70,71].

Moving on, this review also informs that BNT162b2 is associated with increased risks of stroke (ischemic or hemorrhagic), herpes zoster, paresthesia, appendicitis, and lymphadenopathy in some studies. In other studies, however, BNT162b2 is associated with reduced risks of stroke (ischemic or hemorrhagic), cardiac arrest or death, pulmonary embolism, DVT, intracranial hemorrhage, mesenteric thrombosis, other thrombosis events, anemia, acute kidney injury, acute respiratory distress syndrome, and mortality. Assuredly, at least two studies found that BNT162b2 is unassociated with myocardial infarction, arrhythmia, pericarditis, herpes zoster, CVST, thromboembolism (venous or arterial), thrombocytopenia (immune or non-immune), pulmonary embolism, DVT, Bell’s palsy, Guillain-Barré syndrome, seizure, myelitis, encephalomyelitis, and arthritis. Collectively, mixed signals are present only for the potential risk of stroke, herpes zoster, paresthesia, and appendicitis from BNT162b2, warranting more research to clarify (Figure 1).

This review is not without limitations, however. For one, studies were not screened systematically, and data were not analyzed meta-analytically. Although the lack of meta-analysis is a major drawback of this review, the risk and safety patterns identified herein still provide valuable contributions to our current understanding of BNT162b2 vaccine safety. Second, the surveillance studies reviewed herein have limited follow-up time, typically at 28-day following the first and second BNT162b2 dose. Third, the reviewed surveillance studies did not cover certain geographical locations, most notably the South America, Africa, and Australia continents (Table 1). Fourth, the nature of real-world surveillance studies is observational, not randomized; hence, the presence of confounding variables is inevitable. One serious potential confounder is the healthy vaccine effect, which a systematic review has identified in five out of 19 controlled observational studies on the effectiveness of influenza vaccination [72]. Therefore, the possibility of the healthy vaccine bias overestimating vaccine benefits in the observational surveillance studies discussed in this review must not be neglected.

That said, this review offers several strong suits. First, only quality studies with at least 0.1 million BNT162b2-vaccinated participants and at least one unvaccinated control group were included. Second, studies were selected semi-systematically rather than purely via the conventional approach in narrative reviews. Third, over 20 studies were reviewed as of 14 May 2022, which provides a succinct overview of the current, up-to-date literature. Fourth, risk analyses were classified into cardiovascular, herpetic, thrombotic or thrombocytopenic, neurological, mortality, and other miscellaneous adverse events for ease of comprehension. Fifth, this review is arguably the first in the published literature that describes the potential rare adverse events associated with BNT162b2 in sufficient detail.

## 5. Concluding Remarks

Considering the upsurge in vaccine misinformation and disinformation in most countries, communicating vaccine safety is of utmost importance [5]. This is especially true with BNT162b2, a relatively novel mRNA vaccine technology that is also one of the most effective COVID-19 vaccines currently available [73]. To this end, this review has investigated and described the rare adverse events associated with BNT162b2, based on 21 large-scale controlled surveillance studies in North America, Asia, and Europe, finding that the risk of BNT162b2 is extremely minimal. Only myocarditis (or myopericarditis) is consistently associated with BNT162b2, out of all the other numerous cardiovascular, herpetic, thrombotic, thrombocytopenic, neurological, mortality, and miscellaneous adverse events. Conclusively, the risk of BNT162b2 does not outweigh the numerous risks SARS-CoV-2 poses, which should not deter individuals from vaccination.

## Figures and Tables

**Table 1:** Characteristics of large-scale controlled surveillance studies on the association between BNT162b2 and rare adverse events identified via literature search.

Author	Database	Sample Size	Design	Follow-Up Time
Andrews, et al. [24]	National Immunization Management System, UK	1.7 and 15.1 million person-time years in vaccinated and unvaccinated group, respectively	Controlled cohort	Within 28 days after receiving either first or second dose
Barda, et al. [25]	Calit Health Services, Israel	0.88 million people in each vaccinated and unvaccinated group	Controlled cohort	Within 21 days after receiving either first or second dose
Birabaharan, et al. [26]	TriNetX Analytics Network, USA	0.36 million people in each vaccinated and unvaccinated group	Controlled cohort	Within 28 days after receiving either first or second dose
Hippisley-Cox, et al. [27]	National Immunization Management System, UK	9.5 million people	Self-controlled case series	Within 8–28 days of receiving the first dose
Husby, et al. [28]	Danish Vaccination Register and National Patient Register, Denmark	3.5 million vaccinated and 0.21 million unvaccinated people	Self-controlled case series and controlled cohort	Within 28 days after receiving either first or second dose
Hviid, et al. [29]	Danish Vaccination Register and National Patient Register, Denmark	0.10 million vaccinated and 0.13 million unvaccinated people	Controlled cohort	Within 28 days after receiving either first or second dose
Jabagi, et al. [30]	National Health Data System, France	3.9 million people (aged ≥75 years only)	Self-controlled case series	Within 14 days after receiving either first or second dose
Karlstad, et al. [31]	Nationwide Health Registers from Denmark, Finland, Norway, and Sweden	15 million vaccinated and 4.3 million unvaccinated people	Controlled cohort	Within 28 days after receiving either first or second dose
Kerr, et al. [32]	National Health Service, UK	12.6 million people	Self-controlled case series	Within 28 days after receiving the first dose
Klein, et al. [33]	Vaccine Safety Database, USA	6.2 million people	Self-controlled case series	Within 21 days after receiving either first or second dose
Lai, et al. [34]	Hospital Authority, Hong Kong	0.15 million vaccinated and 0.55 million unvaccinated people	Controlled cohort	Within 28 days after receiving the first dose
Li, et al. [35]	Clinical Practice Research Datalink Aurum, UK, and Information System for Research in Primary Care, Spain	3.6 million people	Self-controlled case series	Within 21 days of the first dose
McKeigue, et al. [36]	National Health Service, Scotland	2.7 million doses	Case-crossover	Within 14 days after receiving either first or second dose
Mevorach, et al. [37]	Ministry of Health, Israel	5 million vaccinated and 9.9 million unvaccinated people	Controlled cohort	Within 30 days after receiving the second dose
Patone, et al. [38]	National Immunization Management System, UK	12.1 million people	Self-controlled case series	Within 28 days after receiving the first dose
	National Health Service, Scotland	1.1 million people	Self-controlled case series	Within 28 days after receiving the first dose
Patone, et al. [39]	National Immunization Management System, UK	17 million people	Self-controlled case series	Within 28 days after receiving the first dose
Shasha, et al. [40]	Meuhedet Health Maintenance Organization, Israel	0.23 million in each vaccinated and unvaccinated group	Controlled cohort	Within an average of 22 and 32 days after receiving the first and second dose, respectively
Simpson, et al. [41]	National Health Service, Scotland	0.82 million	Self-controlled case series	Within 28 days after receiving the first dose
Wan, et al. [42]	Hospital Authority, Hong Kong	0.54 million	Nested case-control	Within 42 days after receiving either first or second dose
Wan, et al. [43]	Hospital Authority, Hong Kong	1.96 million	Nested case-control and self-controlled case series	Within 28 days after receiving either first or second dose
Whiteley, et al. [44]	National Health Service, UK	8.7 million vaccinated and 25 million unvaccinated people	Controlled cohort and self-controlled case series	Within 28 days after receiving the first dose

**Table 2:** Association between BNT162b2 and cardiovascular adverse events from large-scale controlled surveillance studies.

	Mevorach et al. [37]	Patone et al. [39]	Karlstad et al. [31]	Husby et al. [28]	Barda et al. [25]	Klein et al. [33]	Jabagi et al. [30]	Whiteleyet al. [44]	Simpson et al. [41]	Patone et al. [38]	Hippisley-Cox et al. [27]	Lai et al. [34]
Myocarditis	RR: 2.35 EC: 1.35 per 0.1 million people⸸	RR: 3.4 EC: 3 per 1 million people ⸶	RR: 2.04 EC: 0.67 per 0.1 million people ⸿	HR: 3.73 EC: 1.3 per 0.1 million people ‡ ⸹	RR: 3.24 EC: 2.7 per 0.1 million people	RR: 3.75 EC: 6.2 per 1 million doses † ⸹	-	-	-	-	-	-
Hemorrhagic stroke	-	-	-	-	-	Non-sig.	Non-sig.	HR: 0.77 ⸘	Non-sig.	RR: 1.24 EC: 60 per 10 million people ⁋	-	-
Ischemic stroke	-	-	-	-	-	Non-sig.	Non-sig.	HR: 0.90 ⸘	-	-	RR: 1.06 EC: 143 per 10 million people	-
Myocardial infarction	-	-	-	-	Non-sig.	Non-sig.	Non-sig.	-	-	-	Non-sig.	-
Arrhythmia	-	-	-	-	Non-sig.	Non-sig.	-	-	-	-	-	-
Pericarditis	-	-	-	-	Non-sig.	Non-sig.	-	-	-	-	-	-
Cardiac arrest or death	-	-	-	HR: 0.51	-	-	-	-	-	-	-	-
Others **	-	-	-	-	-	-	-	-	-	-	-	Non-sig.

Abbreviations/acronyms: EC: excess cases; HR: hazard ratio; RR: risk or relative ratio. Notes: (i) non-sig. means not statistically significant; (ii) if EC is not provided, it is not available; (iii) hyphen (-) denotes not available, that is, unexamined. ⸸ The risk increased to an RR of 8.96 (13.73 excess), 6.13 (9.56 excess), and 3.58 (5.9 excess) in males aged 16–19, 20–24, and 25–29 years, respectively. The risk also increased to an RR of 7.56 (1.89 excess) in females aged 20–24 years. However, the risk was statistically insignificant in females aged 16–24 and >30 years and males aged >30 years. ⸶ The risk is limited to post-second dose in individuals under 40 years only. The risk decreased to an RR of 1.31 (1 excess case per 1 million people) post-first-dose, and was statistically insignificant in individuals over 40 years. ⸿ The risk is limited to males who have received two doses of BNT162b2, which decreased to an RR of 1.4 (0.27 excess) in males who have received only one dose. In females, the RR is 1.46 and 1.25 after the first and second dose, respectively. † The risk is limited to 12–39-year-olds only who have received either BNT162b2 or mRNA-1273. If limited to the second dose only, the risk increased to an RR of 4.07 (10.1 excess). The risk is statistically insignificant in all ages or after the first dose. ⸹ The risk is a composite of myocarditis and/or pericarditis. ‡ The risk is limited to females only. The risk is statistically insignificant in males or 12–39-year-olds. ⁋ The risk is statistically insignificant when limited to males only. However, the risk increased to an RR of 1.44 and 1.84 in females at 1–7- and 14–21-days post-vaccination, respectively. The risk also became statistically insignificant when limited to Scotland residents only instead of the entire UK ⸘ The risk is limited to individuals under 70 years only. In individuals over 70 years, the risk was significant but overlapped substantially with the negative control of lower limb fracture; hence, deemed non-significant in this review. ** The risk is heart failure, microangiopathy, stress cardiomyopathy, arrhythmia, coronary artery disease, or carditis.

**Table 3:** Association between BNT162b2 and herpes infection events from large-scale controlled surveillance studies.

	Barda et al. [25]	Wan et al. [43]	Shasha et al. [40]	Birabaharan et al. [26]
Herpes zoster	RR: 1.43 EC: 15.8 per 100,000 people	iRR: 5.23 ‡ EC: 7 per 1 million doses	Non-sig.	Non-sig.
Herpes simplex virus infection	Non-sig.	-	-	-

Abbreviations/acronyms: EC: excess cases; HR: hazard ratio; iRR, incidence rate ratio; N/A: not available; RR: risk or relative ratio. Notes: (i) non-sig. means not statistically significant; (ii) if EC is not provided, it is not available; (iii) hyphen (-) denotes not available, that is, unexamined. ‡ The risk is limited to within two weeks of the first dose. The risk is similar at the iRR of 5.82 at 3–4 weeks after the first dose and 5.14 at 1–2 weeks after the second dose, but turns insignificant at 3–4 weeks after the second dose.

**Table 4:** Association between BNT162b2 and thrombotic or thrombocytopenic adverse events from large-scale controlled surveillance studies.

	McKeigue et al. [36]	Kerr et al. [32]	Andrews et al. [24]	Hippisley-Cox et al. [27]	Hviid et al. [29]	Simpson et al. [41]	Klein et al. [33]	Whiteley et al. [44]	Barda et al. [25]	Jabagi et al. [30]	Patone et al. [38]
Cerebral venous sinus thrombosis	Non-sig.	Non-sig.	Non-sig.	Non-sig.	Non-sig.	Non-sig.	Non-sig.	-	-	-	-
Thrombocytopenia	-	-	Non-sig.	Non-sig.	Non-sig.	Non-sig.	-	Non-sig.	Non-sig.	-	-
Venous thromboembolism	-	-	-	Non-sig.	-	-	Non-sig.	-	-	-	-
Arterial thromboembolism	-	-	-	Non-sig.	-	Non-sig.	-	-	-	-	-
Pulmonary embolism	-	-	-	-	Non-sig.	-	Non-sig.	HR: 0.78 ‡	Non-sig.	Non-sig.	-
Deep vein thrombosis	-	-	-	-	Non-sig.	-	-	HR: 0.82 ⸸	-	-	-
Intracranial hemorrhage	-	-	-	-	-	-	-	-	RR: 0.48 EC: −2.9 per 100,000 people	-	-
Subarachnoid hemorrhage	-	-	-	-	-	-	-	-	-	-	Non-sig.
Arterial thrombosis	-	-	-	-	Non-sig.	-	-	-	-	-	-
Immune thrombocytopenia	-	-	-	-	-	Non-sig.	Non-sig.	-	-	-	-
Disseminated intravascular coagulation	-	-	-	-	-	-	Non-sig.	-	-	-	-
Portal vein thrombosis	-	-	-	-	-	-	-	Non-sig.	-	-	-
Splanchnic vein thrombosis	-	-	-	-	Non-sig.	-	-	-	-	-	-
Mesenteric thrombosis	-	-	-	-	-	-	-	HR: 0.65 ‡	-	-	-
Intracranial venous thrombosis	-	-	-	-	-	-	-	Non-sig.	-	-	-
Hemorrhagic events	-	-	-	-	-	Non-sig.	-	-	-	-	-
Other venous events	-	-	Non-sig.	-	-	-	-	Non-sig.	-	-	-
Other arterial events	-	-	-	Non-sig.	-	-	-	HR: 0.69 ‡	-	-	-
Other thrombosis events	-	-	-	-	-	-	-	-	RR: 0.46 EC: −2.2 per 100,000 people	-	-

Abbreviations/acronyms: EC: excess cases; HR: hazard ratio; RR: risk or relative ratio. Notes: (i) non-sig. means not statistically significant; (ii) if EC is not provided, it is not available; (iii) hyphen (-) denotes not available, that is, unexamined. ‡ The risk is limited to individuals under 70 years only. In individuals over 70 years, the HR is 0.54 for both pulmonary embolism and mesenteric thrombosis and 0.52 for arterial events. ⸸ The risk is limited to individuals under 70 years only. In individuals over 70 years, the risk is still significant but overlaps substantially with the negative control of lower limb fracture; hence, deemed non-significant in this review.

**Table 5:** Association between BNT162b2 and neurological adverse events from large-scale controlled surveillance studies.

	Wan et al. [42]	Li et al. [35]	Shasha et al. [40]	Barda et al. [25]	Klein et al. [33]	Patone et al. [38]	Lai et al. [34]
Bell’s palsy	Non-sig.	Non-sig.	Non-sig.	Non-sig.	Non-sig.	Non-sig.	Non-sig.
Paraesthesia	-	-	RR: 1.21 EC: 39.5 per 10,000 person-years	Non-sig.	-	-	-
Guillain-Barré syndrome	-	-	Non-sig.	-	Non-sig	Non-sig.	-
Seizure or convulsion	-	-	-	Non-sig.	Non-sig.	-	Non-sig.
Vertigo	-	-	-	Non-sig.	-	-	-
Myelitis	-	-	-	-	Non-sig.	Non-sig.	Non-sig.
Encephalomyelitis	-	-	-	-	Non-sig.	-	Non-sig.
Encephalitis	-	-	-	-	-	Non-sig.	-
Meningitis	-	-	-	-	-	Non-sig.	-
Myasthenic disorder	-	-	-	-	-	Non-sig.	-
Demyelinating events	-	-	-	-	-	Non-sig.	-

Abbreviations/acronyms: EC: excess cases; RR: risk or relative ratio. Notes: (i) non-sig. means not statistically significant; (ii) if EC is not provided, it is not available; (iii) hyphen (-) denotes not available, that is, unexamined.

**Table 6:** Association between BNT162b2 and mortality adverse events from large-scale controlled surveillance studies.

	Hviid et al. [29]	Whiteley et al. [44]	Xu et al. [61]	Husby et al. [28]
Mortality	Non-sig.	HR: 0.24 ‡	-	-
Non-COVID-19 mortality	-	-	RR: 0.41 ⸸	-
Cardiac arrest or death	-	-	-	HR: 0.51

Abbreviations/acronyms: EC: excess cases; HR: hazard ratio; RR: risk or relative ratio. Notes: (i) non-sig. means not statistically significant; (ii) if EC is not provided, it is not available; (iii) hyphen (-) denotes not available, that is, unexamined. ‡ The risk is limited to individuals under 70 years only. In those over 70 years, the HR is 0.19. ⸸ The risk is limited to the post-first dose only. In the post-second dose, the RR is 0.34.

**Table 7:** Association between BNT162b2 and other miscellaneous adverse events from large-scale controlled surveillance studies.

	Barda et al. [25]	Klein et al. [33]	Lai et al. [34]
Appendicitis	RR: 1.4 EC: 5 per 100,000 people	Non-sig.	-
Lymphadenopathy	RR: 2.43 EC: 78.4 per 100,000 people	-	-
Anemia	RR: 0.79 EC: −18.7 per 100,000 people	-	-
Acute kidney injury	RR: 0.44 EC: −4.6 per 100,000 people	-	HR: 0.58
Acute liver injury	-	-	
Pancreatitis	-	-	
Acute respiratory distress syndrome	-	-	HR: 0.21
Neutropenia	Non-sig.	-	-
Lymphopenia	Non-sig.	-	-
Uveitis	Non-sig.	-	-
Arthritis	Non-sig.	-	Non-sig.
Kawasaki disease	-	Non-sig.	-
Thyroiditis	-	-	Non-sig.
Type 1 diabetes	-	-	Non-sig.
Erythema multiforme	-	-	Non-sig.
Chilblain	-	-	Non-sig.
Rhabdomyolysis	-	-	Non-sig.
Syncope	-	-	Non-sig.
Narcolepsy	-	-	Non-sig.

Abbreviations/acronyms: EC: excess cases; HR: hazard ratio; RR: risk or relative ratio. Notes: (i) non-sig. means not statistically significant; (ii) if EC is not provided, it is not available; (iii) hyphen (-) denotes not available, that is, unexamined.

## Data Availability

Not applicable.
